# Evolutionary Overview of Molecular Interactions and Enzymatic Activities in the Yeast Cell Walls

**DOI:** 10.3390/ijms21238996

**Published:** 2020-11-26

**Authors:** Renata Teparić, Mateja Lozančić, Vladimir Mrša

**Affiliations:** Faculty of Food Technology and Biotechnology, University of Zagreb, Pierottijeva 6, 10000 Zagreb, Croatia; rteparic@pbf.hr (R.T.); mlozancic@pbf.hr (M.L.)

**Keywords:** yeast cell wall, cell wall proteome, glucan, mannan, glucanases, transglycosylases

## Abstract

Fungal cell walls are composed of a polysaccharide network that serves as a scaffold in which different glycoproteins are embedded. Investigation of fungal cell walls, besides simple identification and characterization of the main cell wall building blocks, covers the pathways and regulations of synthesis of each individual component of the wall and biochemical reactions by which they are cross-linked and remodeled in response to different growth phase and environmental signals. In this review, a survey of composition and organization of so far identified and characterized cell wall components of different yeast genera including *Saccharomyces*, *Candida*, *Kluyveromyces*, *Yarrowia,* and *Schizosaccharomyces* are presented with the focus on their cell wall proteomes.

## 1. Introduction

Fungal cell walls are composed of glucans (β-1,3-, β-1,6-, β-1,4- and α-1,3-glucans), chitin, and glycoproteins. The basic component of the cell wall moiety in almost all fungal cell walls is the β-1,3-glucan. The composition of the N-linked and O-linked oligosaccharides in cell wall glycoproteins vary, with *Saccharomyces cerevisiae* and *Candida albicans* having high mannan structures and *Sz. pombe* having galactomannans. Schweigkofler et al. [1] analyzed monosaccharide patterns in the cell wall of different fungi and found three main types: (a) the glucose, mannose type; (b) the glucose, mannose, galactose type and (c) the glucose, mannose, galactose, rhamnose type. The glucose, mannose type predominated and it was found in 51 species of the genera *Saccharomyces, Pichia, Candida, Debaryomyces,* and *Kluyveromyces*, with very different mannose proportions ranging from 22% to 75% [1]. Glucose, mannose, galactose type was found in 26 strains within the genera *Pichia, Candida, Arxula, Debaryomyces,* and *Yarrowia* [1]. The proportion of glucose ranged from 28% to 65%, and the proportion of mannose from 18% to 56%. In almost all strains galactose was the least present, ranging from 2% to 27%. The same glucose, mannose, galactose pattern was found in *Sz. pombe* [1]. It is not clear what additional physico-chemical or structural properties have been acquired by the incorporation of galactose in the cell wall but it should be noted that it requires additional enzymes. Glucans, chitin, and glycoproteins are covalently cross-linked by a dynamic process that occurs extracellularly. All fungi contain a similar collection of the cross-linking enzymes capable of creating links among glucan, chitin, and glycoprotein molecules. Hydrolases and transferases are the primary contributors to the cell wall dynamics. The yeast genomes contain a number of genes coding for glucanases and chitinases, often having overlapping functions [2]. The glycosyltransferases have both synthetic and lytic activities, enabling them to cut molecules and then attach them back, building polymers in an environment in which no external source of energy is present. In order to achieve suitable elasticity to allow the growth of the cell wall and budding of cells, the activities of these enzymes must be strictly controlled. Cell wall proteins without known enzymatic activity are considered to have structural functions, to play roles in cell adhesion and mating, or to connect them to other cell wall constituents [3]. Genetic redundancy that exists among many cell-wall associated proteins additionally complicate the determination of the functions of different cell wall proteins. Accordingly, disruptions of single genes hardly ever result in a clear mutant phenotype. Sometimes, phenotypes are detected after the deletion of multiple genes of the same family. In this paper, an overview of enzymes and enzymatic activities that synthesize the carbohydrate part of cell walls of different yeasts is presented. Differences in the presence and type of these enzymes result in different compositions of the carbohydrate moieties of different yeast species. Furthermore, the findings on the composition of the protein part of the cell wall of different yeasts are summarized. The proteins are divided into three basic groups: the group of proteins that are enzymatically active, putatively structural wall proteins, and adhesins. Finally, an overview of current knowledge on wall proteomes of various yeasts is given.

## 2. Composition and Synthesis of Carbohydrate Parts of Yeast Cell Walls

### 2.1. Variations in the Composition of Carbohydrate Parts of Cell Walls of Different Yeasts

The *S. cerevisiae* cell wall consists largely of β-1,3-glucan (50–55%) with lesser amounts of β-1,6-glucan (10–15%) [4]. The wall also contains a small amount of chitin (1–2%). Most of the chitin is found in bud scars formed during the separation of mother and daughter cells during budding but a small amount is also found in lateral cell walls [4]. The β-1,6-glucan is attached to the non-reducing ends of β-1,3-glucan chains participating in the formation of the cell wall matrix [4,5]. The cell wall proteins contain N-linked outer chain mannan with up to 200 mannose residues and short O-linked mannan chains of up to 5 mannose units. GPI (glycosylphosphatidylinositol) anchored proteins are attached to β-1,6-glucans that are connected with the oligosaccharide part of the GPI anchor [6]. Melanin, α-1,3-glucan, and galactomannans are absent from the *S. cerevisiae* cell wall. The yeast wall is organized so that the outer surface of the wall contains large amounts of mannan, i.e., mannoproteins, while glucans and chitin are more concentrated in the layer of the cell wall adjacent to the plasma membrane.

In most aspects, the *C. albicans* cell wall resembles the one of *S. cerevisiae* [7,8]. The major constituents are β-1,3-glucan (30–39%) and β-1,6-glucan (43–53%), with a small amount of chitin (2–6%) [7]. As in *S. cerevisiae*, the β-1,6-glucan is attached to the β-1,3-glucan chains. The wall lacks α-1,3-glucan and galactomannans. During host infection, the *C. albicans* wall contains melanin, which is thought to help strengthen the wall. *C. albicans* synthesizes N- and O-linked mannans attached to the cell wall glycoproteins.

The carbohydrate part of the *Sz. pombe* cell wall is composed of 42% β-1,3-glucan, 2% β-1,6-glucan, 9–14% α-galactomannan, and 18–28% α-1,3-glucan [9]. The cell wall consists of three layers [9] and lacks chitin [10,11]. Furthermore, instead of the branched β-1,6-glucan, it contains short polymers termed diglucan (2%) comprising a backbone of β-1,6-linked glucoses, 75% of which carry β-1,3-linked glucoses [11]. α-Glucan is probably synthesized at the cell membrane, according to the localization of synthase Mok1/Ags1p at the cell membrane [12,13]. Humbel et al. [14] investigated the distribution of β-1,6-glucan, β-1,6-branched β-1,3-glucan, and linear β-1,3-glucan in the cell wall of *Sz. pombe* and found linear β-1,3-glucan exclusively localized in the primary septum of dividing cells. Both β-1,6-branched β-1,3-glucan and β-1,6-glucan form a less dense, central layer of the wall, with β-1,6-glucan located in the outer part of this layer, close to the α-galactomannan layer. The vegetative cell walls lack chitin, but chitin has been found as a component of the conidial cell wall. Melanin is not present in the *Sz. pombe* cell wall. The *Sz. pombe* cell wall proteins have both N-, and O-linked galactomannans [11].

The cell wall of *Kluyveromyces lactis* is similar to this of *S. cerevisiae* with the outer layer formed of mannoproteins and the inner of glucan [15]. β-1,3-glucan builds up to 50% of cell wall dry weight, while chitin contributes with only 1–3% [16]. Contrary to *S. cerevisiae*, whose cell wall density and polysaccharide composition changed considerably when grown at different temperatures, pH, and on different carbon sources [17], *K. lactis* glucan content was according to the authors’ conclusion, not dependent on the carbon source [15]. *S. cerevisiae* cell walls were considerably thicker than those of *K. lactis* when cells were grown on glucose. When *K. lactis* was grown on ethanol, cell wall showed similar thickness to that of *S. cerevisiae* but had a considerably increased sensitivity to Zymolyase speaking in favor of increased β-1,3-glucan concentration. The thickness of the *S. cerevisiae* cell wall does not increase upon growth on ethanol, but it becomes slightly less sensitive to the Zymolyase. Furthermore, *S. cerevisiae* cell wall mannose content increases when cells were grown on ethanol, while the mannose content of *K. lactis* cell wall decreased under the same conditions [15]. Since Zymolyase sensitivity is generally connected with the amount of β-1,3-glucan, walls containing more β-1,3-glucan being more sensitive, results indicate that different yeasts respond differently to environmental changes in terms of cell wall components’ regulation. In this way, they are able to adapt their walls to different environmental influences according to yeasts’ lifestyles.

### 2.2. Enzymatic Activities Involved in the β-1,3-Glucan Synthesis

**Figure 1:**
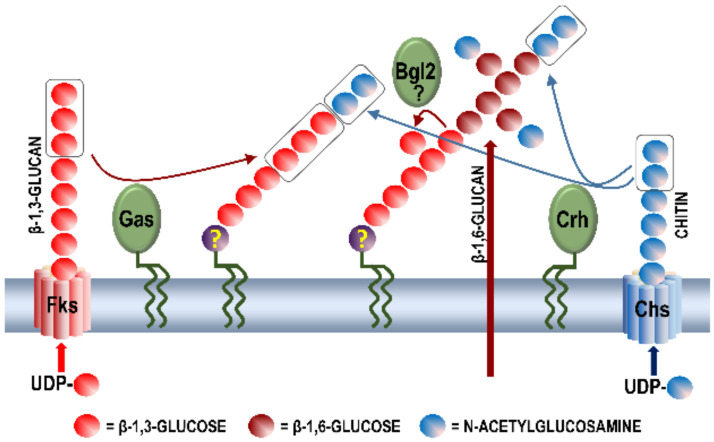
Synthesis of carbohydrate components of the *Saccharomyces cerevisiae* cell walls. Glucan and chitin are synthesized by plasma membrane-associated β-1,3-glucan synthases Fks1-3 and chitin synthases Chs1-3, respectively. Both polymers are simultaneously excreted into the cell wall. Cell wall glycosylphosphatidylinositol (GPI)-anchored enzymes of the Gas and Crh families rearrange wall polysaccharides by transferring parts of protruding β-1,3-glucan and chitin, respectively, to existing β-1,3-, or β-1,6-glucans in the wall. Cell wall non-covalently bound enzyme Bgl2 has a role in branching of β-1,3-glucan chains.

β-1,3-Glucan is the main component of all characterized fungal cell walls that is responsible for the osmotic stability of the cell. It is a branched polymer with branching in the β-1,6-positions making between 30% and 80% of the cell wall mass [5,18]. The β-1,3-glucan helix acts as a coiled spring-like structure giving elasticity and tensile strength to the cell wall [4]. It is synthesized by β-1,3-glucan synthases localized in the plasma membrane, which extrude newly synthesized linear glucan into the cell wall. The enzyme adds glucose residues using cytoplasmic UDP-glucose as the substrate, to the non-reducing end of the growing linear glucan polymer. The polymer is secreted through a channel formed by the enzyme transmembrane domains into the cell wall. *C. neoformans* and *C. albicans* have a single β-1,3-glucan synthase, Fks1, while *S. cerevisiae* has three genes coding for glucan synthases, but Fks1 synthetizes most of β-1,3-glucan during yeast vegetative growth [5]. *Sz. pombe* has four genes coding for β-1,3-glucan synthases, but only one of them has been shown to be involved in septation, mating, polarized growth, and spore formation and germination [19]. Morphology and growth rate of *fks1* mutants are dramatically affected [5]. Fks1 in *S. cerevisiae* co-localizes to areas of polarized growth together with actin patches [20], while in *Sz. pombe* it localizes to the septum [19]. Rho1 protein, a part of the MAP kinase signaling pathway that regulates growth and the cell wall integrity response, acts as a regulatory subunit of Fks1 activating the synthesis of glucan when cells are growing, or during the cell wall stress, and inactivating it when it is not needed [4]. This model of β-1,3-glucan synthesis implies that glucan synthases use an intracellular source of UDP-glucose without the need for UDP-glucose transport across the membrane. Glycosidic linkages are formed within the space created by transmembrane domains of the enzyme by adding glucose units to the reducing end of the chain, while the ready polysaccharide protrudes through the synthase into the surrounding space forming the inner layer of the wall (Figure 1).

Since the β-1,3-glucan molecules consist of very long chains of up to 1500 glucose units, it is difficult to envisage that such long chains could be made without eventually blocking the enzymatic activity of glucan synthase. Indeed, there is evidence that proteins of the Gas family possess the transglucosidase activity and that they transfer portions of the β-1,3-glucan protruding from the glucan synthase to the already synthesized glucan chain, liberating in this way the enzyme for further elongation of the glucan chain (Figure 1). How, or if at all, β-1,3-glucan chains are anchored to the cell membrane is still not known. Speculations that the putative anchor moiety involves a glycolipid or a membrane protein have never been experimentally corroborated. Another opened question is whether β-1,3-glucan chains are somehow covalently cross-linked to form a network similar to the bacterial peptidoglycan. A putative cross-linker should be a bivalent molecule able to connect two glucan chains, thus it could not be a carbohydrate. It has been proposed that some proteins that are covalently attached to glucan can form more than one link, bridging different carbohydrate chains [21]. However, knockouts of corresponding genes did not bring about the expected phenotype. Thus, this question still remains open.

### 2.3. Enzymatic Activities Involved in the β-1,6-Glucan Synthesis

β-1,6-Glucan is a significant element of cell walls of many yeasts including *C. albicans* and *S. cerevisiae* [4,5,22]. In *S. cerevisiae,* it is cross-linked with β-1,3-glucan, chitin, and with the GPI anchored proteins [23,24], playing an important role that is still not completely understood but seems to include interconnecting different wall components [4,5]. However, the β-1,6-glucan synthase has not yet been definitively identified in any fungal species. In *S. cerevisiae* a number of proteins that play roles in the synthesis of β-1,6-glucan have been identified [4], including Kre1, Kre5, Kre6, Kre9, Knh1, Big1, Rot1, and Skn1. Kre5, Big1, and Rot1 proteins are localized in the ER and probably participate in the control of the transport of proteins between ER and Golgi through protein-folding/quality control mechanisms. On the other hand, Kre6 and Skn1 are structurally similar to glycosylhydrolases/transglycosidases and according to that might have a role in cross-linking the β-1,6-glucan with other cell wall components. However, Kre6 and Skn1 are localized in the Golgi, and it is hard to expect that they have a role in cell wall matrix formation. Kre1 is GPI-bonded cell wall protein. The β-1,6-glucan of the *kre1* mutant cell wall is shorter, suggesting that Kre1 functions in the cell wall to elongate β-1,6-glucans [25]. Kre9 and Knh1 are cell wall proteins that have a function in crosslinking β-1,6-glucan into the wall [4]. Simultaneous deletion of *KNH1* and *KRE9* is lethal. It is assumed that these enzymes have a role in the cross-linking of cell wall components or in the folding and targeting of the putative β-1,6-synthase to the plasma membrane. It has been shown that the β-1,6-glucan is synthesized at the plasma membrane [26], thus it is expected that the β-1,6-synthase will have a mechanism similar to the β-1,3-glucan and chitin synthases. β-1,6-Glucans have not been found in the cell wall of the fungi *Aspergillus fumigatus* [27] and *Neurospora crassa* [28], indicating that the role of β-1,6-glucan in the cell wall of different fungal species is not universal.

### 2.4. Enzymatic Activities Involved in Chitin Synthesis

Yeast species generally contain 1–2% of the cell wall mass made of chitin, while filamentous fungi may have up to 15% of chitin in their cell walls. Plasma membrane-associated chitin synthases use cytosolic UDP-N-acetylglucosamine as a substrate to synthesize linear polymers of β-1,4-N-acetylglucosamine by the addition of N-acetylglucosamine to the non-reducing end. Chitin is simultaneously excreted into the cell wall through multiple transmembrane domains of the chitin synthases forming a channel, reducing end first (Figure 1). In the cell wall, chitin chains are bonded by hydrogen bonding and assemble into microfibrils [7]. Extruded chitin can be deacetylated by chitin deacetylases to generate chitosan. Chitin polymer significantly contributes to the cell wall integrity, and disturbed chitin synthesis results in deformed and osmotically unstable yeast cells. All characterized fungi have chitin synthases and most have multiple genes encoding chitin synthases. Most frequently, one or two of the chitin synthases synthesize the majority of chitin, while others produce only smaller quantities [29,30]. *S. cerevisiae* has three chitin synthases: Chs1, Chs2, and Chs3 [31]. The Chs3 chitin synthase generates 80–90% of chitin [32] in the wall including chitin covalently cross-linked to β-1,3-glucan and the chitin ring formed during bud formation [24]. The *chs3* mutant has defects in cell wall integrity and highly reduced levels of chitin, as well as very slow growth. Chs1 replenish chitin lost during cytokinesis, and Chs2 has a role in the formation of the primary septum. The simultaneous deletion of *CHS1*, *CHS2,* and *CHS3* results in a lethal phenotype [33]. *C. albicans* has four [34] and *Sz. pombe* two chitin synthase genes [35], one of which lacks synthase activity [36]. Deletions of the major chitin synthases in *C. albicans* result in a weakened cell wall and reduced growth rate. In contrast, deletion of *chs1* and *chs2* in *Sz. pombe* does not affect growth rate and chitin was not detected in the *Sz. pombe* vegetative cell wall [37]. However, the Chs1 is required for the asci formation, while the Chs2 is involved in the septum formation [35,38]. Generally, biosynthesis of β-1,3-glucan and chitin share the same cell strategy and it can be assumed that the corresponding enzymatic mechanisms are used for the synthesis of other fungal wall polysaccharides, as well.

## 3. Yeast Cell Wall Proteomes

The development of bioinformatics and genome sequencing enabled much faster and easier identification and characterization of cell wall proteins. A number of potential GPI-anchored cell wall proteins were identified by bioinformatic methods [39,40], and by mass spectrometry [3,37,41]. Mass spectrometry was applied on the cell wall proteins isolated by chitinase and glucanase and digested by trypsin, or on the proteins isolated by “trypsin shaving” in which cell walls were trypsinized without previous isolation of the proteins and the released tryptic fragments were subjected to mass spectrometric analysis. Lately, glycosidic bonds of glycoproteins were digested by trifluoromethanesulfonic acid to isolate the proteins from the cell wall, followed by trypsinization and nano-LC/MS/MS analysis of peptides obtained in this way [42]. The trifluoromethanesulfonic acid treatment allows to identify positions of N-glycosylation since the first glucosamine bonded to the asparagine is not released by this treatment, and tryptic peptides can be identified with the attached glucosamine. The O-glycosylation sites can be detected by NH_4_OH treatment of the cell wall proteins that removes the oligosaccharide and tags the attachment site with -NH_3_ groups [43]. Tagged proteins are then trypsinized and analyzed by mass spectrometry to identify the peptides containing the -NH_3_ tag. *S. cerevisiae* cell wall proteins belonging to glycosylhydrolase families 16, 17, and 72, proteins of the Pir (protein with **i**nternal **r**epeat) family, and adhesins/flocculins have homologs in almost all sequenced fungal genomes. Such results indicate that they have general roles indispensable for yeast cells and have thus been evolutionary conserved. Proteins detected in the cell walls of different yeasts by proteomic investigations are summarized in Table 1.

Bioinformatic comparison of genes coding for 187 proteins involved in the biosynthesis of cell walls of different yeast strains/genera indicated that the variability of cell wall proteomes correlated with the taxonomic distance of the compared proteomes [44,45]. As cell walls of all yeasts share the same structural principles, it can be assumed that the proteins involved in the biosynthesis and remodeling of wall polysaccharides were rather conserved throughout the evolution. On the other hand, cell walls are also involved in the communication of the cell with its surrounding. For this purpose, yeasts need a more variable set of proteins that would reflect different environmental conditions and lifestyles.

### 3.1. Cell Wall Proteins with Enzyme Activities

A major part of the so far known cell wall proteins is thought to have a role in attachments and rearrangements of various elements of the wall. Some of them function as glycosylhydrolases and others as transglycosidases that synthesize new glycosidic bonds between the cleaved polymers [46,47]. Genes coding for these enzymes are conserved in fungal genomes. In most cases, these enzymes are encoded by multiple genes organized in families providing the redundancy of cross-linking activity and ensuring the functionality of the cell wall even if one of the genes is functionally lost by mutation. Indeed, genetic analyses show that in most cases the loss of a single enzyme does not result in a significant change in phenotype. Sometimes, the result is a decreased growth rate or increased sensitivity to cell wall perturbation agents. More apparent phenotype usually occurs in multiple mutants in which more or all members of the gene/protein family are deleted.

Enzymes of the Gas/Phr family are among the best characterized cell wall enzymes. These GPI-anchored enzymes are capable of rearranging β-1,3-glucan [47] and generating non-reducing ends in β-1,3-glucan for attachment of β-1,6-glucosylated mannoproteins [48]. *S. cerevisiae* has five *GAS* genes [49], *C. albicans* five homologous *PHR* genes [50], *K. lactis* cell wall proteome comprises three homologs of *S. cerevisiae* Gas proteins, KlGas1, KlGas3, and KlGas5 [15], and *Sz. pombe* has four *GAS* genes [51] having overlapping specificity. In *S. cerevisiae*, Gas1 and Gas5 are expressed in vegetative cells, while Gas2 and Gas4 are sporulation-specific [52]. Deletion of *GAS1* results in enlarged spherical cells, increased quantity of β-1,3-glucan secreted to the medium, and reduced level of glucan in the cell wall [53]. Single *GAS2* or *GAS4* deletion has no effect on sporulation, but *gas2gas4* double deletion results in the severely disturbed formation of the spore cell wall, indicating that their activities are redundant [49,54]. In *C. albicans,* Phrl and Phr2 are required for normal morphology and virulence and their expression is pH-dependent, with Phr2 being expressed at acidic and Phr1 at neutral to alkaline pH [48]. Phr1 is required for the adhesion to epithelial cells and abiotic substrates [50]. According to that the consequence of deletion of the encoding genes is pH-conditional defects in cell morphology and virulence [48]. In *Sz. pombe*, Gas4 is essential for maturation of ascospore wall, and Gas1 and Gas5 are most probably involved in elongation of β-1,3-glucan chains [51].

The Crh/Utr family has been identified as one of the major cell wall proteins family in the cell walls of *S. cerevisiae* and *C. albicans*. This family of transglycosidases has a role in the addition of short chitin chains to the non-reducing ends of β-1,6-glucan [46], and β-1,3-glucan [55] in *S. cerevisiae* and *C. albicans*. *S. cerevisiae* has two homologs, Crh1 and Crh2 [46] and *C. albicans* has three enzymes, Crh11, Crh12, and Utr2 [55]. Crh11 and Crh12 are the homologs of *S. cerevisiae* proteins Crh1 and Crh2 [46,56]. Utr2 seems to be a β-glucanase included in filamentation, adherence, and virulence [57]. *K. lactis* cell wall proteome contains Crh1 (KlChr1) and Utr2 (KlUtr2) as well [15]. Utr2 is found in *K. lactis* cell wall exclusively in logarithmic growth phase on either glucose or lactose or ethanol. In the cell wall of *Y. lipolytica,* Hwang et al. [58] found two cell wall proteins YlCrh1 and YlCrh2 showing high amino acid sequence similarity to *S. cerevisiae* Crh1 and Crh2. Deletion of the *YlCRH1* and *YlCRH2* resulted in increased sensitivity to Congo red and Calcofluor white. The YlCrh1 and YlCrh2 showed to be able to degrade β-1,3 glycosidic linkage. The YlCrh1 protein showed to be capable to hydrolyze β-1,4- and β-1,6-linkages as well, although with lower specific activities than that on β-1,3-linkage. Both YlCrh1 and YlCrh2 cannot degrade the substrate of exo-β-1,3-glucanases (4-nitrophenyl-β-D-glucopyranoside), indicating that they are endo β-1,3-glucanases [58].

Members of Bgl2/Scw4/Scw10/Scw11 family of enzymes homologous to plant β-1,3-glucanases have been found in *S. cerevisiae* and *C. albicans* cell walls. *S. cerevisiae* and *C. albicans* Bgl2 cleave glucose units from the reducing end of the β-(1,3)-oligosaccharide, transferring enzyme-bound oligosaccharide to an acceptor β-(1,3)-oligosaccharide, either at C-6 of the non-reducing end or at C-6 of an internal glucose unit [59]. The *S. cerevisiae bgl2* mutant has higher level of chitin in the wall [60]. Deletion of *bgl2* in *C. albicans* results in a disturbed cell wall and lower virulence [61]. In contrast to *C. albicans* and *S. cerevisiae,* Bgl2 homologs were not found in the *Sz. pombe* cell wall. *S. cerevisiae* single *scw4* and *scw10* mutants are sensitive to cell wall destabilizing agents like Calcofluor white and Congo red, and the *scw4scw10* mutant shows a synergistic effect [62]. Grbavac et al. [63] demonstrated that Scw4 undergo double processing, by yapsins and Kex2 protease. Processing at the yapsin site significantly lowers the potential for covalent attachment of Scw4 to glucan. Furthermore, the overproduction of a fully processed form of Scw4 leads to high mortality, particularly in the stationary phase of growth, and to markedly increased cell size. The physiological role of Scw4 and Scw10 are still unknown. Two additional members of this family, Scw1 and Mp65, were detected in the cell walls of *C. albicans*. Castillo et al. [64] detected Scw1 protein in the cell wall of *C. albicans* that lacks a GPI motif, and it possesses glucanase activity. It has been proposed that the formation of disulfide bridges might be a mechanism of the binding Scw1 to the cell wall [64]. *K. lactis* cell wall proteome contains Scw4 (KlScw4) protein as well [15].

*S. cerevisiae* cell wall proteins Dfg5 and Dcw1 are homologs of *N. crassa* α-1,6-mannanases Dfg5 and Dcw1. Maddi and Free [28] established that in the *N. crassa* cell wall the α-1,6-mannose core of N-linked galactomannan was required for the covalent binding of cell wall proteins. Dfg5 and Dcw1 in this fungi cleave the N-linked α-1,6-mannan of mannoproteins and create new glycosidic bonds with cell wall glucans, so the *dfg5dcw1* double mutant is unable to incorporate cell wall proteins [65]. According to that, it has been speculated that the functions of Dcw1 and Dfg5 in *S. cerevisiae* cell wall might also be related to the transfer of GPI-proteins from the membrane to the cell wall [66]. However, Dfg5 and Dcw1 in *S. cerevisiae* are shown to be crucial for the formation of the cell wall in growing buds [67], but the exact enzymatic functions of Dcw1p and Dfg5p are still unknown. *Dfg5* mutants of *C. albicans* are shown to be defective in hypha formation [68]. Dfg5 and Dcw1 have overlapping functions in *S. cerevisiae* and *C. albicans*, as well as in *N. crassa* [65,66,67,68].

*S. cerevisiae* has three and *C. albicans* four chitinase genes [69,70]. In *S. cerevisiae,* Cts1 functions in degrading chitin synthesized between the mother and daughter cells as the primary septum [71], so in *cts1* mutant, the division of mother and daughter cells is affected. In *C. albicans*, deletion of Cht3 results in a similar cell separation phenotype [70], while endochitinase 2, Cht2, might have a role in cell separation after cytokinesis since it was shown to be located close to the wall that separates daughter from mother cell [61]. Colussi et al. [72] characterized *K. lactis* chitinase KlCts1p. They showed that disruption of *CTS1* resulted in a separation defect phenotype and that KlCts1p could restore normal morphogenesis to *S. cerevisiae cts1* mutant cells, indicating identical roles of these two proteins.

Generally, this short overview clearly points out the complexity and variability of enzymatic activities present in the yeast cell wall. Enzymes that are capable of splitting and creating new glycosidic bonds are quite numerous, have different specificities, and are fairly conserved among different yeasts. Notably, for some of them, activities have only been demonstrated in vitro, or not at all, so that their exact physiological function still remains unclear. Yet, it can be assumed that they contribute to the pronounced plasticity and flexibility of the yeast cell wall.

### 3.2. Adhesins

Adhesins are crucial for biofilm formation and cell adhesion, allowing pathogenic fungi to start the infection process, or saprophytic fungi to stick to their nutrient-rich substrates. A number of adhesins have been identified in fungal cell walls. *S. cerevisiae* produces a- and α-agglutinin during mating that enables cell–cell interactions [73]. The a-agglutinin is expressed by mating-type cells in response to mating pheromone and consists of two subunits, Aga1 and Aga2. The carboxyl terminus of Aga2 has a high affinity for binding of α-agglutinin. The α-agglutinin, Sag1, is expressed in mating-type **α** cells and binds to Aga2 through its N-terminal region. *S. cerevisiae* also has five *FLO* genes four of which are coding for lectin-like proteins (Flo1, Flo5, Flo9, Flo10) that bind to mannose and/or glucose [74] enabling cell adhesion and flocculation. Flo11 on the other hand has a role in biofilm formation and recognizes a variety of other substrates [75] like agar and plastic surfaces, probably facilitating adhesion to plant materials in nature. *K. lactis* cell wall proteome contains a homolog of Flo5 (KLLAOE14586g) and two isoforms of Muc1/Flo11, while *S. cerevisiae* contains only one form of that protein [15]. KlFlo5 is preferentially found during growth in the presence of lactose. KlMuc1a predominates in the logarithmic phase and KlMuc1b in the stationary phase. Furthermore, KlMuc1a is more represented upon growth on glucose, while KlMuc1b seems to be lactose-induced.

In *Sz. pombe*, the Map4 protein facilitates cell–cell interactions during mating, and no other adhesins were found [76]. *C. albicans* cell wall contains different types of proteins such as adhesins and lectins, that allow attachment of the cells to different surfaces, and different enzymes involved in the degradation of the protective structures of host tissues or involved in the synthesis of the cell wall itself. Some of the proteins are bound in the cell wall non-covalently, whereas others are covalently bound to other cell wall proteins or to different cell wall polysaccharides [5,7]. Cell adhesion has been widely investigated in *C. albicans* as a human pathogen [74]. It was found that *C. albicans* genome contained a family of eight *ALS* genes coding for adhesins [77], among which Als1 and Als5 were demonstrated to bind a broad range of peptides [78], while Als3 had a crucial role in the biofilm formation and can also facilitate binding to host tissues [79] but is not required for virulence [80]. Als1 and Als10/Als2 were shown to be involved in epithelial adhesion and invasiveness due to their adhesion properties [81]. *C. albicans* Hwp1 adhesin enables adhesion to human skin and oral epithelia and has a role in the formation of biofilms [82].

### 3.3. Cell Wall Proteins with still Unidentified Functions

Cell wall proteins without any apparent enzyme activities have been identified in a number of yeast species suggesting that they might have non-enzymatic, or structural functions. It has to be noted, however, that the designation of these proteins as “structural” merely relates to the fact that no other function has been attributed to them so far. This group of proteins shows lower levels of homology across the yeast species and genera than the cell wall enzymes. Most of them are attached to the cell wall glucan through the GPI remnants. Since the GPI anchor plays a vital role in the transport of GPI-anchored proteins through the secretory pathway, mutations in genes encoding the proteins required for the biosynthesis of the GPI anchor are lethal in *S. cerevisiae* [40,83] and in *Sz. pombe* [84], while in *C. albicans* biosynthesis of GPI anchor is vital for normal morphology and virulence [85].

The structural cell wall protein most broadly represented among yeast genera is Ecm33. Mutants lacking Ecm33 induce the cell wall integrity (CWI) pathway and show altered cell wall architecture in both *S. cerevisiae* [86], *Sz. pombe* [87] and *C. albicans* [88]. In *S. cerevisiae* deletion of *ECM33* results in spherical and swollen cells, highly sensitive to Calcofluor white and Congo red [86,89]. Ecm33 is found to be essential for normal cell wall architecture and integrity, and for the function and expression of some cell surface proteins in *C. albicans* [88,89]. *ecm33* mutants of *C. albicans* have swollen and spherical cells highly sensitive to cell wall-perturbing agents [86,89]. There are two *ECM33* paralogues in the genome of *Sz. pombe* (Meu10 and Ecm33). Ecm33 has yet unidentified but critical role in keeping the integrity of *Sz. pombe* cell wall [87].

Furthermore, *S. cerevisiae* cell wall GPI proteins comprise members of the TIR family (Tir1-Tir4, Tip1, Cwp1, Cwp2, and Dan1-Dan4) [90], FIT family (Fit1-Fit3) [91], yapsins family [92,93], and proteins Spi1, Sed1, Ccw12, and Ccw14. The physiological functions of these proteins are still mostly unknown. Cwp1 and Cwp2 are repressed while the other members of the TIR family are induced under anaerobic conditions [94]. *tir1*, *tir3,* and *tir4* mutants under anaerobic conditions show growth defects. Members of the FIT family possibly have a role in binding iron from the medium [91], while Spi1 and Sed1 were proposed to have a protective role under glucose limitation [95]. The roles of Ccw12 and Ccw14 are still unknown. It was shown that *ccw12* mutant has increased mortality in the stationary phase of growth [96,97]. Yapsins are GPI-anchored family of five aspartyl proteases (Yps1-3, Yps6, Yps7) [92,93] with substrate specificity similar to the Kex2 protease. A mutant lacking all five yapsins has a considerably reduced quantity of β-1,3- and β-1,6-glucan in the cell wall and undergoes lysis at 37 °C [98].

Proteomic analysis of hyphal and yeast form cells of *C. albicans* shows that there are some protein differences between these two cell types [42,99,100]. Castillo et al. [64] found 21 cell wall surface proteins in *C. albicans* proteome containing a cell wall signal in their immature form. Proteins Als10, Bgl21, Pga30, Pga31, Pga45, Phr2, Rbt1, and Utr2 were identified in *C. albicans* cell wall for the first time in this investigation [64]. Out of the 21 identified proteins by Castillo et al. [64], only Bgl21 and Scw1 were found not to contain a GPI-binding domain, while all the others do. Proteins Als1, Pga24, Pga30, Pga45, Rbt1, and superoxide dismutase Pga2 were identified with a single peptide, indicating that these proteins are present in the cell wall in smaller quantities. Pga2, Pga4, Pga24, Pga29, Pga30, Pga31, and Pga45 are predicted GPI proteins of the same family [39,101]. Pga2 is a superoxide dismutase, homologous to *S. cerevisiae* Yjr104c, with a function in ROS removal. Pga24 and Pga29 are proteins with unidentified roles. Pga30 is similar to the *S. cerevisiae* Sed1, and Pga45 shows a low similarity to a glucan 1,4-α-glucosidase Yil169c. Pga31 is assumed to be a membrane protein with low similarity to glucan 1,4-α-glucosidase and exo-α-sialidase [102]. Ssr1 might be a structural protein, without catalytic function found so far [101].

In the cell wall of *Sz. pombe,* six covalently bound cell wall proteins were identified by tandem mass spectrometry, including two alkali-extractable (Psu1 and Asl1) and four GPI-bonded cell wall proteins (Gas1, Gas5, Ecm33, and Pwp1) [37]. Pwp1 is an abundant structural GPI-bonded protein with the still unknown role. Psu1 is a homolog of the *S. cerevisiae* Sun family, while Asl1 has no homologs in *S. cerevisiae. Sz. pombe* cells with deleted *PSU1* are resistant to β-1,3-glucanase digestion and unable to complete cell division [103]. Although the overall quantity of proteins in the cell walls of *Sz. pombe* is slightly lower, the level of their glycosylation is significantly lower than that in *C. albicans* and *S. cerevisiae*.

The cell wall proteome of *K. lactis* shares several features with the wall proteomes of *S. cerevisiae*, *C. albicans,* and *C. glabrata*. They have a similar number of covalently linked proteins [97], and their wall proteome composition depends on the growth conditions. Furthermore, the number of GPI proteins in the wall is much bigger than the number of Pir proteins. *K. lactis* cell wall proteome contains some species-specific proteins with the yet unknown role (KLLA0E24959g, KLLA0E24893g and KLLA0B14498g) but comprises some homologs of those of *S. cerevisiae* including yapsins (KlYps1, KlYps7 and KLLA0D01507g-homolog of ScYps3), Ccw14 (KlCcw14), and Cwp1 (KlCwp1a and KlCwp1b) as well [15]. Putative cell wall proteins in *K. lactis*, such as KLLA0E24959g, KLLA0E24893g, and KLLA0B14498g, lack a counterpart in the yeast databases. Potential GPI cell wall protein KLLA0E24893g with the unidentified role and with no apparent homolog in *S. cerevisiae* is exclusively found upon growth on ethanol. KLLA0B14498g and KLLA0E24959g are found to be more represented in the walls upon growth on glucose. In contrast, KlCcw14 seems to be ethanol-specific [15]. Although *S. cerevisiae* usually displays a higher degree of redundancy in enzymatic functions than *K. lactis* [104], *K. lactis* contains two isoforms of Cwp1, while *S. cerevisiae* contains only one form of that protein. The GPI protein KlCwp1a possesses a Pir repeat as well as its homolog ScCwp1, while KlCwp1b does not, suggesting that KlCwp1a can bind through its GPI anchor to β-1,6-glucan and through its Pir repeat to β-1,3-glucan [105,106]. Both isoforms of Cwp1 in *K. lactis* are predominantly found in the stationary phase.

*Yarrowia lipolytica* grows as a combination of short mycelial and yeast-like cells and has a high capacity to metabolize lipids and hydrocarbons to secrete heterologous proteins and to accumulate large amounts of organic acids. This made it interesting for industrial application and for the production of heterologous proteins. However, relatively little is known about its cell wall structure, especially about its cell wall proteins. Ramon et al. [107] characterized Ywp1 protein to be covalently linked to the mycelial cell wall, although it does not contain specific Pir- or GPI- binding motifs. Ywp1 is neither O-glycosylated nor N-glycosylated [108]. According to the results obtained by Ramon et al. [107], one part of Ywp1 is bound covalently directly to the glucan network or by disulfide bridges to other cell wall proteins, and other part that is bound to β-mercaptoethanol-extractable N-glycosylated high molecular weight proteins. Deletion of *YWP1* has no effect on the cell growth rate, morphology, or the general structure of the cell wall. Furthermore, Jaafar and Zueco [109] identified Ylcwp1 cell wall protein containing a putative signal peptide and GPI-attachment signal and some other structural features, suggesting that *YlCWP1* encodes a GPI-cell wall protein of *Y. lipolytica*.

Pir protein family has been found in *S. cerevisiae* and *C. albicans*, as well as in many other budding yeasts, but not in *Sz. pombe*. Pir proteins possess Kex2 cleavage sites, multiple internal repeats, and a cysteine domain at their C-terminus [103]. The connection between the protein and β-1,3-glucan is in the form of an alkali sensitive glutamate–glucose ester linkage [105]. Mutants lacking single Pir proteins do not have severe cell wall defects. The quadruple *S. cerevisiae pir1pir2pir3pir4* mutant has larger cells and shows increased susceptibility to cell wall perturbing reagents [21]. The Pir proteins have been shown to be responsible for *S. cerevisiae* resistance against a plant antifungal compound osmotin and are important for the survival of cells in the stationary growth phase [110,111]. Pir proteins comprise several potential binding sites for β-1,3-glucan [112], so they might have a role in crosslinking β-1,3-glucan chains and fortifying cell wall upon under stress [113]. This presumption is additionally corroborated by the finding that in *C. albicans*, which contains only one Pir protein, its role seems to be essential [114]. *K. lactis* cell wall proteome contains two Pir proteins (KlPir1a and KlPir1b) [15]. Jaafar et al. [115] characterized *Y. lipolytica* cell wall protein Ylpir1. Ylpir1 is the homolog of Pir4 cell wall protein of *S. cerevisiae*. Disruption of *YlPIR1* resulted in slightly increased resistance to Zymolyase, Calcofluor white, and Congo red, but growth rate and cell morphology were not affected by this mutation [115].

## 4. Conclusions

The cell wall as the yeast’s outermost cellular structure defines cell shape, protects it from environmental stresses, and allows interactions of the cell with its environment. Some of these functions like the preservation of cell integrity and osmotic stability are universal and require concomitant activities of enzymes that have to be conserved in all fungi. Others are more species-specific and reflect different cell habitats and lifestyles. These characteristics require more specific cell wall enzymes/proteins, as well. In order to understand how exactly yeasts form their external armor and how this structure can withstand high osmotic pressures and still be flexible enough to allow growth, budding, and other cellular events, future investigations should reveal the physiological roles of cell wall proteins in more details. Besides, our comprehension of the variability of the cell wall composition and understanding of the molecular basis of cell wall integrity is crucial for the development of new biotechnological applications of fungi as well as the creation of new antifungal therapies. Examples of such applications are the use of antifungal drugs based on the inhibition of β-1,3-glucan synthesis [116,117], or expression of heterologous proteins directed to the cell wall and displayed at the cell surface [118], but further strategies applied in cell surface engineering could result in new biotechnology tools with various applications.

## Figures and Tables

**Table 1:** Proteins isolated identified in cell walls of different yeasts.

	*Saccharomyces cerevisiae*	*Candida albicans*	*Schizosaccharomyces pombe*	*Kluyveromyces lactis*	*Yarrowia lipolytica*
**Cell wall proteins with enzyme activities**					
**Glucan transferases**	Gas1, Gas2, Gas3, Gas4, Gas5	Pga4, Phr1, Phr2	Gas1, Gas4, Gas5	KlGas1, KlGas3, KlGas5	-
**Glucanase homologs**	Bgl2, Scw4, Scw10	Bgl21, Scw1, Scw4, Mp65	-	KlScw4	-
**Chitin transferases**	Crh1, Crh2	Crh11, Crh12, Utr2	-	KlCrh1, KlUtr2	YlCrh1, YlCrh2
**Chitinases**	Cts1, Cts2, Cts3	Cht1, Cht2, Cht3, Cht4	-	KlCts1p	-
**Mannanase homologs**	Dfg5, Dcw1	Dfg5, Dcw1	-	-	-
**Adhesins**					
**Cellular interactions**	Aga1, Aga2, Sag1, Flo1, Flo5, Flo9, Flo10, Flo11	Als1, Als2, Als3, Als5, Hwp1	Map4	KlMuc1a, KlMuc1b, KlFlo5	-
**Proteins with unidentified functions**					
**Proteins linked to glucan through Pir-sequences**	Pir1, Pir2, Pir3, Pir4	Pir1	-	KlPir1a, KlPir1b	Ylpir1
**GPI-anchored proteins**	Ecm33, Ccw12, Ccw14, Sed1, Tir1-Tir4, Tip1, Cwp1, Cwp2, Dan1-Dan4, Fit1-Fit3, Spi1, Yps1-3, Yps6, Yps7	Ecm33, Pga24, Pga29, Pga30, Pga45,÷Ssr1/Ccw14, Rbt1	Ecm33, Meu10, Pwp1	KlEcm33, KlCcw14, KlCwp1a, KlCwp1b, KLLA0E24959g, KLLA0E24893g, KLLA0B14498g, KlYps1, KlYps7 KLLA0D01507g	Ylcwp1
**Proteins with unknown linkage**			Psu1, Als1		Ywp1
